# Off-label use catalogue of tumor anti-angiogenic drugs in China: a narrative review

**DOI:** 10.3389/fphar.2025.1668620

**Published:** 2025-10-14

**Authors:** Difei Yao, Yangmin Hu, Shanshan Weng, Tiantian Wang, Tao Zhu, Lulu Liu, Huan Luo, Wei He, Haibin Dai

**Affiliations:** ^1^ Department of Pharmacy, Second Affiliated Hospital of Zhejiang University School of Medicine, Hangzhou, China; ^2^ Research Center for Clinical Pharmacy, Zhejiang University, Hangzhou, China; ^3^ Department of Medical Oncology, Second Affiliated Hospital of Zhejiang University School of Medicine, Hangzhou, China

**Keywords:** anti-angiogenic therapy, off-label use, drug label, guidelines, drug management

## Abstract

**Background:**

The management of off-label use (OLU) is an important aspect of standardizing and promoting the rational use of antitumor drugs. Tumor anti-angiogenic therapy (AAT) is essential in cancer treatment. However, OLU of anti-angiogenic drugs is common in clinical practice. Recognizing and standardizing the OLU of AAT is a significant challenge that needs to be addressed in the clinic.

**Aim:**

We aim to collect and categorize the OLU of AAT in oncology based on clinical guidelines, providing practical guidance for drug management.

**Methods:**

We established a multidisciplinary expert team to screen and include evidence-based OLU information.

**Results:**

By organizing the OLU information, it is evident that these 14 drugs of AAT have indications for 33 cancer types (36 items). Among them, 12 (85.7%) have OLU recommendations, totaling 215 items. These OLU recommendations were classified into four major categories. Cancer type is the most common OLU category, with 12 drugs covering 64 cancer types and 155 items. In addition, OLU in therapy lines, regimens, and dosage was identified in 3–4 drugs each. Bevacizumab, the drug most frequently involved in OLU, is associated with 12 cancer types, 3 therapy lines, 6 regimens, and 2 dosage-related OLU items. In addition, lenvatinib, pazopanib, sorafenib, apatinib, and sunitinib also have OLU recommendations for more than five cancer types.

**Conclusion:**

Our work offers an updated reference for the OLU of tumor AAT and highlights the need for further exploration into specific management measures for OLU in clinical practice.

## Introduction

Off-label use (OLU) of medications, also known as the unregistered use of a drug, occurs when a drug is prescribed for indications, dosages, treatment courses, routes of administration, or patient populations that fall outside the scope of its approved label as authorized by drug regulatory authorities (Maschke, 2018). In recent years, China has implemented a series of policies to regulate the OLU of anti-tumor drugs. The *Law for Licensing Medical Practitioners of the People’s Republic of China (P.R.C.)*, originally enacted in 1999, was amended in 2021. This amendment marked the first legal protection for evidence-based off-label drug use in China. From 2018 to 2024, the National Health Commission (NHC) of the P.R.C. has issued annual guidelines for the clinical application of novel anti-tumor drugs, incorporating indications approved in other countries or regions. In 2021, the NHC released the *Administrative Measures for the Clinical Application of Anti-Tumor Drugs (Trial)* and the *Guiding Principles for the Clinical Application of New Anti-Tumor Drugs* to further standardize the management of off-label drug use. Scholars have also issued guidelines for off-label drug use recommendations, which primarily encompass general guidelines and those specifically tailored for pediatric or ophthalmic medications (Meng et al., 2022a; Li et al., 2022; Zuo et al., 2022; Singh et al., 2024; Meng et al., 2022b). Additionally, professional societies in China, such as the Guangdong Pharmaceutical Association, Zhejiang Pharmaceutical Association, Shandong Pharmaceutical Association, Sichuan Pharmaceutical Association, and Medical Association, have published several directories and expert consensuses on off-label drug use (Shi et al., 2024; International Medical Society and Breast Cancer Group Branch of Oncologist Chinese Medical Doctor Association, 2022), providing valuable references for the OLU of drugs. However, among these catalogs, one catalog with the most comprehensive inclusion of tumor anti-angiogenic therapy (AAT) lists a total of 19 off-label items for 7 drugs. Another catalog includes nine items for five drugs of tumor AAT. The remaining catalog does not include any drug for tumor AAT.

AAT represents a critical class of anticancer agents, which is widely used in clinical practice (Liu et al., 2023). Due to its unique mechanism of action, AAT can be effectively combined with cytotoxic drugs and tumor immunotherapy agents, offering broad application potential (Li and Fang, 2024). By October 2024, 14 drugs of AAT had been approved for use in China. However, the approved indications for drugs of AAT in China are fewer than those recommended by international guidelines, leading to the frequent OLU in real-world settings (Fontanals et al., 2023; Levêque, 2016). In clinical practice, identifying and managing the OLU of AAT remains challenging. One of the primary reasons is the slow progress in establishing hospital-level systems for documenting and approving OLU. Additionally, the lack of up-to-date reviews and summaries of OLU evidence further complicates this issue (Shi et al., 2024).

The Oncology Pharmacy Group of the Hospital Pharmacy Committee of the Zhejiang Pharmaceutical Society has issued a series of consensus statements on the off-label use of specific drugs of AAT, such as recombinant human endostatin, anlotinib, pazopanib, and regorafenib in China. However, with the rapid accumulation of new clinical evidence and the increasing number of AAT drugs entering the market, the existing off-label consensus requires updating. To address this need, we established a multidisciplinary expert writing group to systematically classify and summarize the evidence for 14 drugs of tumor AAT. This initiative aims to provide an updated and comprehensive reference for the clinical OLU of AAT in oncology.

### Aim

In this narrative review, we screened and collected the most recent drug label information and guideline recommendations for the use of 14 drugs of AAT in oncology. These findings were systematically organized and summarized by drug, with a particular focus on OLU details, recommended sources, and the corresponding levels of evidence.

## Methods

### Data sources

Two investigators independently collected drug names, drug labels, and guideline documents according to the following data collection steps. After completion, a third investigator compared and verified the completeness of the collected data.

We identified 14 AAT drugs approved and marketed in China before October 2024 through the National Medical Products Administration (NMPA) database (https://english.nmpa.gov.cn/database.html). For each drug, we obtained the latest prescribing information (current as of October 2024) from China (https://db.yaozh.com/instruct), the United States (https://www.fda.gov/drugs), and the European Union (https://www.ema.europa.eu). Details of drug label versions/dates are provided in Supplementary Table S1.

We collected the latest tumor treatment guidelines (current as of October 2024). The primary guidelines included the following: Chinese Society of Clinical Oncology (CSCO) guidelines (https://www.csco.org.cn), National Comprehensive Cancer Network (NCCN) guidelines (https://www.nccn.org), and oncology diagnostic and therapeutic guidelines issued by the China National Health Commission (China, 2023).

### Guideline and drug label inclusion/exclusion criteria

**FIGURE 1:**
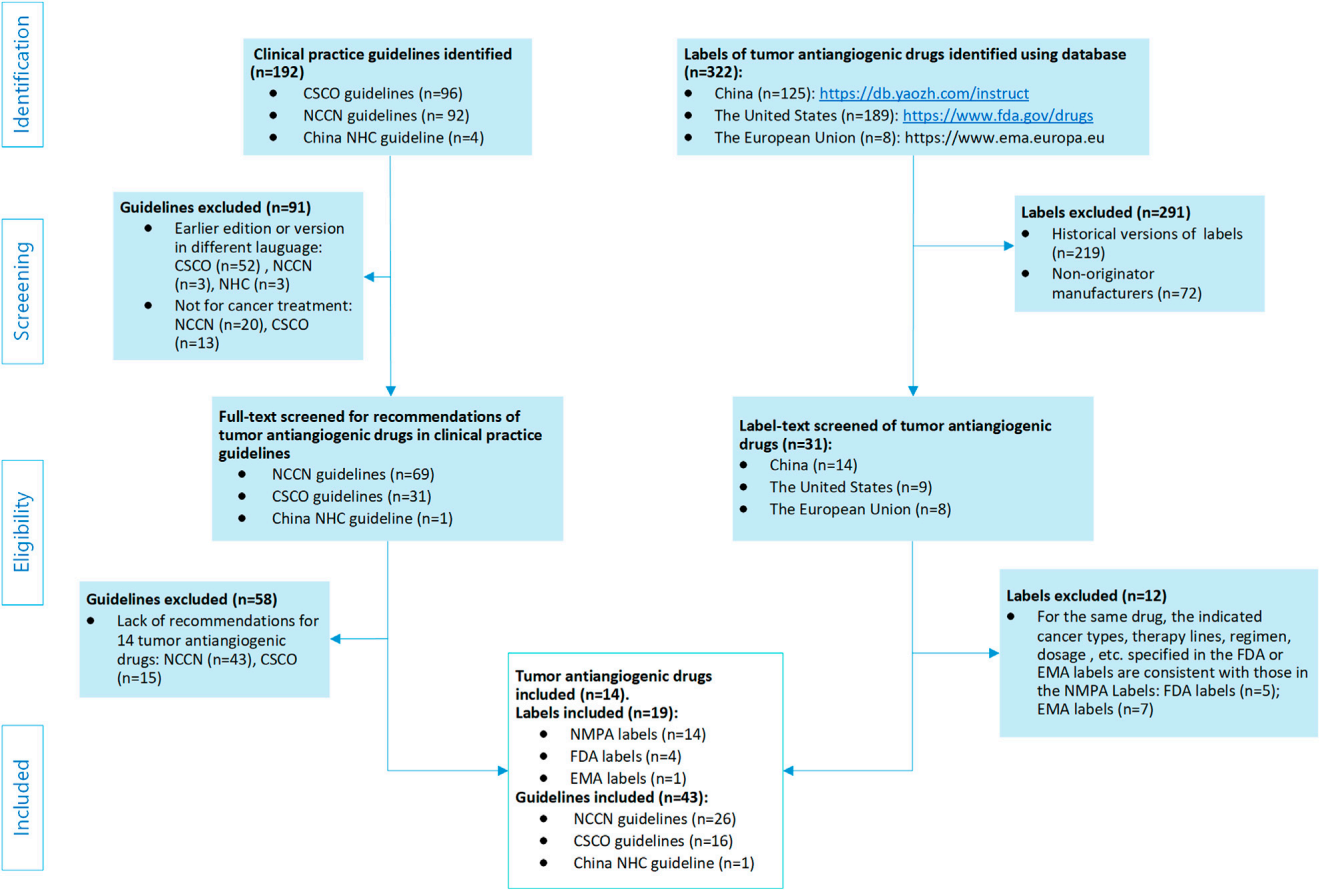
Flowchart of the guideline and drug-label screening process. NMPA, P.R.C National Medical Products Administration; FDA, U.S. Food and Drug Administration; EMA, European Medicines Agency; CSCO, Chinese Society of Clinical Oncology; NCCN, National Comprehensive Cancer Network; NHC, China National Health Commission.

Inclusion criteria were as follows: NMPA labels for the 14 drugs of AAT available at that time; FDA and EMA labels in cases where discrepancies existed in indications, treatment lines, regimens, or dosage, compared with the NMPA labels; and guidelines that formally recommended any of the 14 AAT drugs as oncology treatment regimens. Exclusion criteria were as follows: drug labels from non-originator manufacturers; historical versions of labels; FDA and EMA product information consistent with the NMPA labeling; and guidelines with recommendations not involving OLU of the 14 AAT drugs. The full process of the guideline and label search is shown in Figure 1.

### Off-label use categorization framework

Two independent investigators evaluated all collected data using an abstraction form (Supplementary Table S2). OLU was defined as meeting any of the following criteria compared with NMPA-approved indications: cancer type(s) not included in the approved indications; treatment lines differing from the approved sequence; combination therapy regimens not specified in the label; dosage regimens deviating from the approved prescribing information.

**FIGURE 2:**
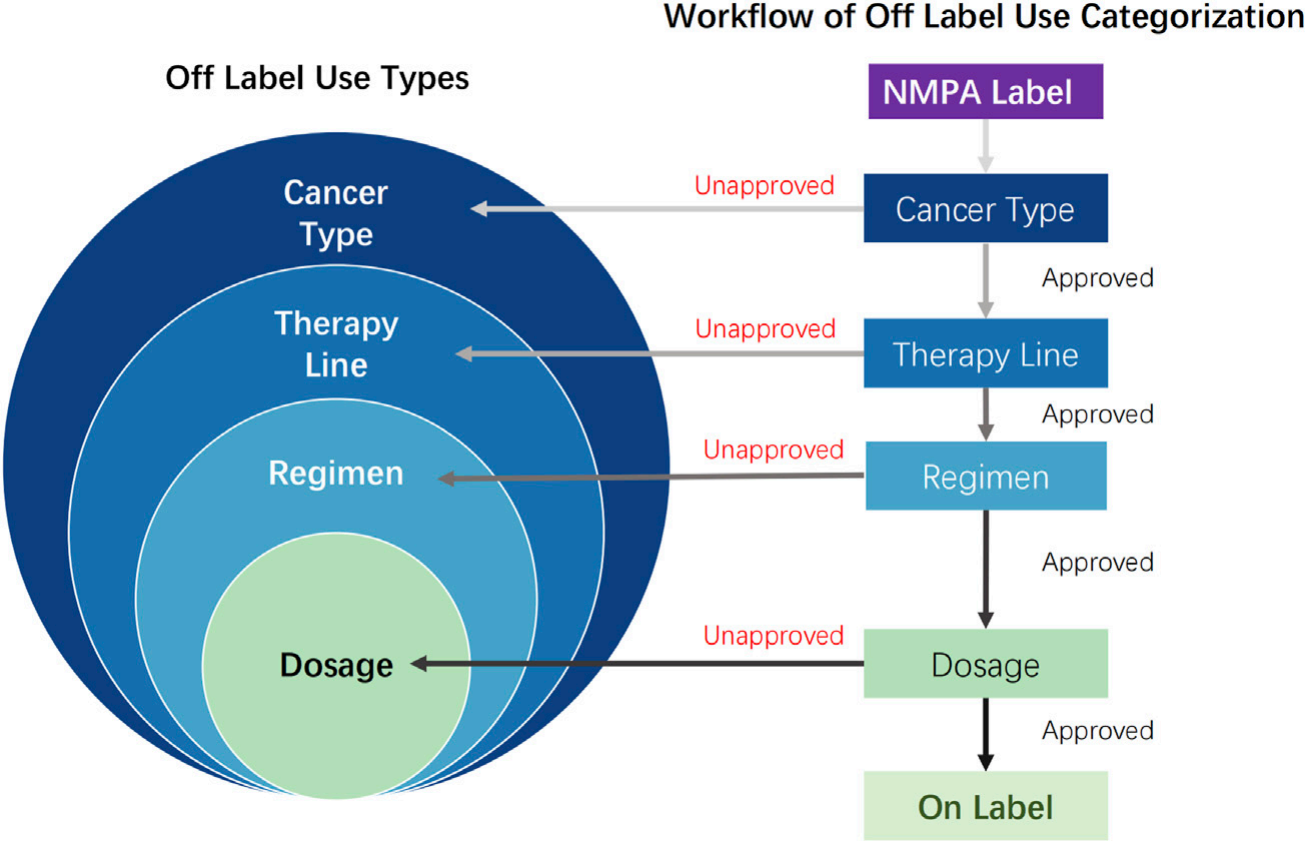
Off-label use types and workflow of off-label use categorization: off-label use cases were hierarchically categorized based on the scope of divergence (broad to narrow), cancer type (unapproved indication), therapy line (unapproved treatment sequence and on-label cancer type), regimen (unapproved combination therapy, on-label cancer type, and therapy line), and dosage (unapproved dosing, on-label cancer type, therapy line, and regimen). NMPA, P.R.C National Medical Products Administration; approved, refers to content described in the NMPA drug label; unapproved, content not included in the NMPA drug label.

As shown in Figure 2, identified OLU cases were hierarchically categorized based on the scope of divergence (broad to narrow): cancer type (unapproved indication), therapy line (unapproved treatment sequence), regimen (unapproved combination therapy), and dosage (unapproved dosing). Each case was assigned exclusively to the most comprehensive applicable category. Dosage OLU implied on-label cancer type, therapy line, and regimen. Regimen OLU implied on-label cancer type and therapy line. Therapy line OLU implied on-label cancer type. This hierarchical classification ensured mutually exclusive categorization across all OLU types. Python 3.12 software was used to perform a kappa agreement test on the OLU classifications of the two investigators. For OLU classification items with disputes, the final category was determined through joint discussion by the article writing team in accordance with the initial workflow criteria.

Evidence classification and recommendation grading adhered to the original guideline standards. When an OLU entry has recommendations from different guidelines, we list all the recommendations. Where recommendations lacked explicit evidence levels, we applied the CSCO evidence classification system. For a quick understanding of the classification of recommendations and evidence, we compiled a table (Supplementary Table S3) that outlines the corresponding recommendation categories and evidence levels. For detailed information, please check the official websites.

## Results

Initially, we identified 192 oncology clinical guidelines and 322 drug product information documents. After multi-round screening (Figure 1), we finally included 19 AAT drug labels [14 NMPA labels (ROCHE, 2024; ChiaTaiTianqing, 2024; JiangsuHengrui, 2023; Pfizer, 2024; Hutchison, 2023; Eisai, 2022; EliLilly, 2022; Hutchison, 2024; SuzhouZelgen, 2022; Novartis, 2021; Bayer, 2021; Bayer, 2018; PFIZER, 2022; Simcere, 2020) and 5 AAT labels from other regions (Roche, 2022; GENENTECH, 2022; PF, 2024; EISAI, 2024; ELILILLY, 2024; NOVARTIS, 2024; CPPI, 2024)], 3 non-AAT labels (MERCK, 2024; Innovent, 2023; ASTRAZENECA, 2023), and 43 guidelines (NCCN, 2024b; NCCN, 2024d, NCCN, 2024p, NCCN, 2024v; CSCO, 2023c, NCCN, 2024o; CSCO, 2023h; CSCO, 2023f, NCCN, 2024l, NCCN, 2024k; NCCN, 2025b; CSCO, 2023e, NCCN, 2024r, NCCN, 2024s; CSCO, 2023b; NCCN, 2025c, NCCN, 2024w, NCCN, 2024n; CSCO, 2024e; CSCO, 2024a; NCCN, 2024e, NCCN, 2024q; CSCO, 2023a; CSCO, 2024b; CSCO, 2024c; CSCO, 2023g; CSCO, 2021; CSCO, 2022a; NCCN, 2024h; NCCN, 2024i, NCCN, 2024t, NCCN, 2024u; CSCO, 2022b; NCCN, 2024f; NCCN, 2025a; NCCN, 2024g; CSCO, 2023d, NCCN, 2024j; NCCN, 2024c; CSCO, 2024d; NCCN, 2024a, NCCN, 2024m) for OLU evaluation. After aggregation, the tumor types across the indications of the 14 drugs of tumor AAT were summed, resulting in a total of 33 tumor types involving 36 entries. Among the 14 drugs, 12 had OLU recommendations, with a total of 215 OLU entries determined (Cohen’s kappa = 0.917) (Supplementary Table S4, where each row, except NMPA labels in the table, is considered one entry or item).

### Off-label use of vascular endothelial growth factor inhibitors

Currently, there are two anti-vascular endothelial growth factor (VEGF) antibody drugs available: bevacizumab, approved by the U.S. Food and Drug Administration (FDA) in 2004, and aflibercept, approved in 2011 (Hida et al., 2024). Bevacizumab and aflibercept were approved for marketing in China in 2010 and 2013, respectively. Bevacizumab is widely used in oncological treatments, while aflibercept is primarily indicated for ophthalmological conditions in China (Hang et al., 2023).

In the field of antitumor therapy, the main OLU of bevacizumab involves indications (Table 1). There are 12 cancer types and 57 items involving bevacizumab recommended by guidelines that are not included in the NMPA-approved indications. Additionally, OLU related to treatment regimens includes 22 entries across 6 therapy lines in 4 cancer types. Exceeding the recommended therapy lines was observed in the palliative treatment of three cancer types. Furthermore, differences in dosing practices for specific indications across geographical regions have resulted in variations in the recommended dosages specified in product inserts.

**TABLE 1:** Off-label use of vascular endothelial growth factor and receptor inhibitors.

Drug name Dosage form Strength	Off-label type	Cancer type Subtype (​​molecular subtyping) [NMPA label content]	Therapy line(s)^1^	Regimen	Limiting condition	Evidence and recommendation category
Vascular endothelial growth factor inhibitor						
Bevacizumab solution for infusion 100 mg; 400 mg	NMPA indications	Colorectal cancer		+ Fluorouracil-based chemo		NMPA label (ROCHE, 2024)
		Cervical cancer		+ Paclitaxel/cisplatin + Paclitaxel/topotecan		
		Epithelial ovarian, fallopian tube, or primary peritoneal cancer	First	+ Carboplatin/paclitaxel and maintenance bevacizumab		
		Glioblastoma	Recurrent		Adult	
		Hepatocellular carcinoma	First	+ Atezolizumab		
		Non-small-cell lung cancer	First	+ Platinum-based chemo	Non-squamous	
	Cancer type	Ampullary adenocarcinoma	First	+ Capecitabine/oxaliplatin + Fluorouracil/leucovorin/irinotecan + Fluorouracil/leucovorin/oxaliplatin	Intestinal type	2A, NM (NCCN, 2024b)
				+ Fluorouracil/leucovorin/irinotecan/oxaliplatin	ECOG 0–1	
				+ Capecitabine + Fluorouracil/leucovorin	ECOG 2	
			≥Second	+ Fluorouracil/leucovorin/irinotecan	Prior oxaliplatin-based therapy	
				+ Capecitabine ± oxaliplatin + Fluorouracil/leucovorin/oxaliplatin + Fluorouracil/leucovorin ± irinotecan	ECOG 2	
		Breast Cancer	First	+ Paclitaxel		EMA label (Roche, 2022)
			First	+ Capecitabine	Other chemo unsuited; taxanes and anthracyclines not used in 12 months	
		Central nervous system cancer		Single	Pediatric	2A, UCC (NCCN, 2024p)
		Intracranial and spinal ependymoma		Single	Adult, not subependymoma	2A, OR (NCCN, 2024d)
		Medulloblastoma	Recurrent	+ Temozolomide/irinotecan	Adult	2A, OR (NCCN, 2024d)
		Meningiomas		Single + everolimus		2A, OR (NCCN, 2024d)
		Miscellaneous		Single	Neurofibromatosis type 2 vestibular schwannomas with hearing loss	2A, OR (NCCN, 2024d)
		Astrocytoma (*IDH*-mutant) Oligodendroglioma (*IDH*-mutant, 1p19q codeleted)		Single	WHO grade 3, KPS ≥60	2A, preferred (NCCN, 2024d)
				+ Carmustine + Lomustine + Temozolomide	WHO grade 3, KPS ≥60	2A, OR (NCCN, 2024d)
		Endometrial carcinoma	First	+ Carboplatin/paclitaxel		2A,OR (NCCN, 2024v); NM, Ⅱ (CSCO, 2023c)
			≥Second	Single		2A, OR (NCCN, 2024v); NM, Ⅲ (CSCO, 2023c)
		Malignant sex cord-stromal tumors		Single	Recurrent	2A, OR (NCCN, 2024o); 3, Ⅲ(CSCO, 2023h)
		Melanoma				
		Cutaneous, acral	First	+ (Paclitaxel or albumin-bound paclitaxel) ± platinum		2A, Ⅱ (CSCO, 2023f)
			Second	+ (Paclitaxel or albumin-bound paclitaxel) ± platinum		2A, I (CSCO, 2023f)
		Uveal		+ (Paclitaxel or albumin-bound paclitaxel) ± platinum		2A, Ⅱ (CSCO, 2023f)
		Mucosal		+ (Paclitaxel or albumin-bound paclitaxel) ± platinum		2A, I (CSCO, 2023f)
				+ Atezolizumab		2A, Ⅲ (CSCO, 2023f)
		Mesothelioma				
		Pleural	First	+ (Cisplatin or carboplatin)/pemetrexed	Epithelioid	1, preferred (NCCN, 2024L)
				+ (Cisplatin or carboplatin)/pemetrexed	Biphasic or sarcomatoid	1, OR (NCCN, 2024L)
			≥Second	+ (Cisplatin or carboplatin)/pemetrexed	Nivolumab/ipilimumab first-line	2A, preferred (NCCN, 2024L)
		Peritoneal	First	+ Cisplatin/pemetrexed	Epithelioid	2A, preferred (NCCN, 2024k)
				+ Cisplatin/pemetrexed	Biphasic or sarcomatoid	2A, OR (NCCN, 2024k)
			≥Second	+ Cisplatin/pemetrexed	Nivolumab/ipilimumab first line	2A, preferred (NCCN, 2024k)
				+ Atezolizumab	Immune checkpoint inhibitor-naïve	2A, OR (NCCN, 2024k)
		Renal cell carcinoma		+ Interferon alfa		FDA label (GENENTECH, 2022)
		Clear cell	≥Second	Single		2B, UCC (NCCN, 2025b)
		Non-clear cell		+ Everolimus + Erlotinib	Not collecting duct and medullary regions	NM, Ⅲ (CSCO, 2023e)
				+ Erlotinib	Papillary subtype	2A, OR (NCCN, 2025b)
				+ Everolimus		2A, UCC (NCCN, 2025b)
		Small-bowel adenocarcinoma		+ Fluorouracil/leucovorin/oxaliplatin + Capecitabine/oxaliplatin + Fluorouracil/leucovorin/irinotecan	Intensive therapy suitable	2A, NM (NCCN, 2024r)
			First	+ Fluorouracil/leucovorin/irinotecan/oxaliplatin	Intensive therapy suitable	2A, NM (NCCN, 2024r)
			First	+ Fluorouracil/leucovorin + Capecitabine	Intensive therapy unsuitable	2A, NM (NCCN, 2024r)
		Soft tissue sarcoma				
		Angiosarcoma		Single		2A, UCC (NCCN, 2024s)
		Solitary fibrous tumor		+ Temozolomide		2A, preferred (NCCN, 2024s); 3, Ⅲ (CSCO, 2023b)
		Vaginal cancer: squamous cell carcinoma and adenocarcinoma	First	+ (Cisplatin or Carboplatin)/paclitaxel/pembrolizumab	PD-L1-positive	2A, preferred (NCCN, 2025c)
				+ (Cisplatin or carboplatin)/paclitaxel		2A, preferred (NCCN, 2025c)
				+ Topotecan/paclitaxel		2A, OR (NCCN, 2025c)
			≥Second			2A, OR (NCCN, 2025c)
		Vulvar cancer		+ Cisplatin/paclitaxel		2A, preferred (NCCN, 2024w)
				+ Carboplatin/paclitaxel		2B, preferred (NCCN, 2024w)
	Therapy line	Colorectal cancer [NMPA label: 1st]	First to second	+ Fluoropyrimidine–irinotecan-based chemo + Fluoropyrimidine–oxaliplatin-based chemo	First-line bevacizumab-containing regimen	FDA label (GENENTECH, 2022)
		Epithelial ovarian, fallopian tube, or primary peritoneal cancer [NMPA label: 1st]	≤Third	+ Paclitaxel + PEGylated liposomal doxorubicin + Topotecan	Platinum-resistant recurrence	FDA label (GENENTECH, 2022) EMA label (Roche, 2022)
			Recurrence maintenance	Single	No *BRCA1/2* mutation, bevacizumab in platinum-sensitive recurrence therapy, complete or partial response achieved	2A, Ⅱ (CSCO, 2023h)
			Platinum-resistant recurrence	+ Gemcitabine		2A, Ⅰ (CSCO, 2023h); 2A, OR (NCCN, 2024o)
				Single or + cyclophosphamide (oral)		2A, preferred (NCCN, 2024o); 2B, Ⅲ (CSCO, 2023h)
				+ Carboplatin/gemcitabine or + carboplatin/liposomal doxorubicin		2A, OR (NCCN, 2024o); 2A, Ⅱ (CSCO, 2023h)
				+ Cyclophosphamide (oral)/pembrolizumab		2A, OR (NCCN, 2024o)
				+ Ixabepilone		2B, OR (NCCN, 2024o)
				+ Mirvetuximab soravtansine-gynx	FRα-expressing	2A, UCC (NCCN, 2024o); 2A, Ⅲ (CSCO, 2023h)
		Non-small-cell lung cancer				
		Non-squamous [NMPA label: 1st]	Maintenance	Single	PS 0–2	1, NM (NCCN, 2024n)
				+ Atezolizumab	PS 0–2, PD-L1 ≥1%, prior atezolizumab/carboplatin/paclitaxel/bevacizumab	
				+ Pemetrexed	PS 0–2, prior bevacizumab/pemetrexed/platinum	2A, NM (NCCN, 2024n)
		Non-squamous (*EGFR* ex19del or L858Rm) [NMPA label: 1st]	≥Second	+ Erlotinib	*T790*M-, prior erlotinib/bevacizumab	2A, NM (NCCN, 2024n)
				+ Platinum-based dual-drug chemo	Multiple lesion progression on EGFR-TKI	2A, Ⅰ (*T790*M- or prior third-generation TKI) or Ⅱ (*T790*M+) (CSCO, 2024e)
				+ Single-drug chemo	PS 0–2, prior EGFR*-*TKI and dual-drug chemo	2A, Ⅱ (CSCO, 2024e)
		Non-squamous (*ALK* rearrangement) [NMPA label: 1st]	≥Second	+ Platinum-based dual-drug chemo	Multiple lesion progression	2A, Ⅰ (prior ALK-TKI) or Ⅱ (CSCO, 2024e)
				+ Single-drug chemo	PS 0–2, prior ALK-TKI and dual-drug chemo	2A, Ⅱ (CSCO, 2024e)
		Non-squamous (*ROS1* rearrangement) [NMPA label: 1st]	Second	+ Platinum-based dual-drug chemo	Multiple lesion progression	2A, Ⅰ (CSCO, 2024e)
			Third	+ Single-drug chemo	PS 0–2	2A, Ⅱ (CSCO, 2024e)
	Regimen	Cervical cancer [NMPA label: + paclitaxel/cisplatin or + paclitaxel/topotecan]	First	+ Pembrolizumab/chemo	PD-L1-positive (CPS ≥1)	FDA label (MERCK, 2024)
				+ Carboplatin/paclitaxel		2A, preferred (NCCN, 2024o) 2A, Ⅰ (CSCO, 2023h)
		Colorectal cancer (microsatellite stability or microsatellite instability-low/mismatch repair proficient) [NMPA label: + 5-FU-based chemo]	First	+ Trifluridine pyrimidine	Intensive therapy unsuitable	2B, Ⅲ (CSCO, 2024a)
			≥Third	+ Trifluridine pyrimidine	Prior oxaliplatin and irinotecan	1A, I (CSCO, 2024a); 2A, NM (NCCN, 2024e; NCCN, 2024q)
		Epithelial ovarian, fallopian tube, or primary peritoneal cancer				
		[NMPA label + carboplatin/paclitaxel]	Primary	+ Docetaxel/carboplatin and maintenance bevacizumab	Stage II–IV	2A, OR (NCCN, 2024o)
				+ Docetaxel/oxaliplatin and maintenance bevacizumab	Stage II–IV	2A, UCC (NCCN, 2024o)
				+ Fluorouracil/leucovorin/oxaliplatin + Capecitabine/oxaliplatin	Mucinous carcinoma, stage II–IV	2B, preferred (NCCN, 2024o) 2B, Ⅲ (CSCO, 2023h)
				+ Chemo, maintenance bevacizumab	Clear-cell carcinoma, stage Ⅲ–IV	2A, Ⅱ (CSCO, 2023h)
		Homologous recombination deficiency positive [NMPA label: single]	First-line maintenance	+ Olaparib	Bevacizumab in primary therapy, complete or partial response achieved	FDA label (ASTRAZENECA, 2023)
				+ Niraparib (olaparib-intolerant)		2A, NM (NCCN, 2024o)
		[NMPA label: + carboplatin/paclitaxel]	Platinum-sensitive recurrence	Single		2A, preferred (NCCN, 2024o)
				+ Carboplatin/gemcitabine + Carboplatin/liposomal doxorubicin		2A, preferred (NCCN, 2024o); 1A, NM (CSCO, 2023h)
				+ Niraparib		2B, OR (NCCN, 2024o); 2A, Ⅲ (No *BRCA1/2* mutation) (CSCO, 2023h)
				+5-FU/LV/oxaliplatin or + capecitabine/oxaliplatin		2B, UCC (NCCN, 2024o)
				+ Mirvetuximab soravtansine-gynx	FRα-expressing	2B, UCC (NCCN, 2024o)
		Non-small-cell lung cancer: non-squamous [NMPA label: + platinum-based chemo]	First	+ Erlotinib	*EGFR*-activating mutation	EMA label (Roche, 2022) 2A, UCC (NCCN, 2024n)
				+ Carboplatin/paclitaxel/atezolizumab	PD-L1 ≥ 1%, PS 0–2	1, OR (NCCN, 2024n)
			≥Second	+ Pemetrexed/cisplatin/sintilimab	First- or second-generation EGFR-TKI resistance with *T790*M- or third-generation EGFR-TKI resistance	NMPA label (Innovent, 2023) 1A, Ⅲ (CSCO, 2024e)
	Dosage	Colorectal cancer [NMPA label: 5 mg/kg q2w or 7.5 mg/kg q3w]		10 mg/kg q2w		FDA label (GENENTECH, 2022); EMA label (Roche, 2022)
				15 mg/kg q3w		EMA label (Roche, 2022)
		Non-small-cell lung cancer [NMPA label: 15 mg/kg q3w]		7.5 mg/kg q3w	Non-squamous	EMA label (Roche, 2022)
Vascular endothelial growth factor receptor inhibitors						
Anlotinib capsules 8 mg 10 mg 12 mg	NMPA indications	Non-small-cell lung cancer	≥Third		*EGFR* mutations or *ALK*-positive	NMPA label (ChiaTaiTianqing, 2024)
		Soft tissue sarcoma	≥Second		Prior to anthracycline-based chemo	
		Small-cell lung cancer	≥Third			
		Thyroid carcinoma: medullary			Symptomatic or progression	
		Thyroid carcinoma: differentiated			Refractory radioactive iodine	
	Cancer type	Biliary tract cancers	Second	+ anti-PD-1/PD-L1 therapy	PS ≤ 1	2B, Ⅲ (CSCO, 2023a)
		Esophageal and esophagogastric junction: squamous cell carcinoma	≥Second		PS 0–2	2A, Ⅱ (CSCO, 2024b)
		Renal cell carcinoma: clear cell	First	Single	Poor or intermediate risk	NM, Ⅲ (CSCO, 2023e)
Apatinib tablet 250 mg 375 mg 425 mg	NMPA indication	Gastric or gastroesophageal: junction adenocarcinoma	≥Third	850 mg qd, Single	Prior ≥2 systemic chemo	NMPA label (JiangsuHengrui, 2023)
		Hepatocellular carcinoma	First	250 mg qd, + camrelizumab		
			≥Second	750 mg qd, single	Prior ≥1 systemic therapy	
	Cancer type	Esophageal and esophagogastric junction squamous cell carcinoma	≥Second	250–500 mg, qd	PS 0–2	3, Ⅱ (CSCO, 2024b)
				250 mg qd, + camrelizumab	PS 0–2	3, Ⅲ (CSCO, 2024b)
		Head and neck cancer: adenoid cystic carcinoma			Symptomatic, rapidly progressing	2A, Ⅱ (CSCO, 2024c)
		Melanoma: acral	First	+ Camrelizumab/temozolomide	No brain metastases	2A, Ⅱ (CSCO, 2023f)
				+ Camrelizumab/temozolomide	Unresectable brain metastases, PS 0–2	2A, Ⅱ (CSCO, 2023f)
		Nasopharyngeal carcinoma	≥Second	250 mg qd + camrelizumab	Anti-PD-1-/PD-L1-based therapy naïve	2B, Ⅲ (CSCO, 2023g)
		Ovarian epithelial carcinoma	Recurrence	250 mg qd + doxorubicin liposomes	Platinum-resistant	2A, Ⅱ (CSCO, 2023h)
		Thyroid cancer: differentiated		500 mg qd, single	*RET*-based or unknown, iodine refractory, symptomatic, rapidly progressing	1A, Ⅱ (CSCO, 2021)
	Regimen	Hepatocellular carcinoma [NMPA label: 750 mg qd, single]	Second	250 mg qd + camrelizumab	Prior oxaliplatin-based therapy, Child–Pugh A or B (score ≤7)	2A, Ⅱ (CSCO, 2022a)
Axitinib tablet 1 mg 5 mg	NMPA indication	Renal cell carcinoma	≥Second	Single		NMPA label (Pfizer, 2024)
	Cancer type	Head and neck cancer				
		Adenoid cystic carcinoma		Single	No surgery or radiotherapy option	2A, Ⅱ (CSCO, 2024c)
			First	+ Avelumab	No surgery or radiotherapy option	2B, UCC (NCCN, 2024h)
		Salivary gland tumor		Single	No surgery or radiotherapy option	2B, UCC (NCCN, 2024h)
		Melanoma: mucosal	First	+ Toripalimab		2A, Ⅱ (CSCO, 2023f)
		Soft tissue sarcoma: alveolar soft part sarcoma		+ Pembrolizumab		2A, preferred (NCCN, 2024s); 3, Ⅱ (CSCO, 2023b)
	Therapy line	Renal cell carcinoma				
		Clear cell [NMPA label: subsequent]>	First	+ Avelumab or + pembrolizumab		FDA label (PF, 2024)
				+ Toripalimab	Poor or intermediate risk	1A, Ⅱ (CSCO, 2023e)
				Single		2B, UCC (NCCN, 2025b)
				Single	Favorable or intermediate risk	2A, Ⅱ (CSCO, 2023e)
		Non-clear cell [NMPA label: subsequent]	First	Single		2A, UCC (NCCN, 2025b) NM, Ⅲ (CSCO, 2023e)
				+ Pembrolizumab		NM, Ⅲ (CSCO, 2023e)
	Regimen	Renal cell carcinoma: clear cell [NMPA label: single]	Second or Third	+ Pembrolizumab	Prior TKI therapy	2B, Ⅱ (CSCO, 2023e)
Fruquintinib capsule 1 mg 5 mg	NMPA indication	Colorectal cancer	≥Third	5 mg qd D1-21 q4w	Prior 5-FU, oxaliplatin, and irinotecan-based chemo and prior or not suitable for anti-VEGF therapy and anti-EGFR therapy (*RAS* wild type)	NMPA label (Hutchison, 2023)
Lenvatinib capsule 4 mg 10 mg	NMPA indication	Hepatocellular carcinoma		8–12 mg qd, single		NMPA label (Eisai, 2022)
		Thyroid cancer: differentiated		24 mg qd, single	Radioactive iodine-refractory	
	Cancer type	Endometrial carcinoma	Recurrent	20 mg qd, + pembrolizumab	MMR-proficient or not MSI-H	FDA label (EISAI, 2024)
		Melanoma				
		Cutaneous	≥Second	+ Pembrolizumab	Prior anti-PD-1-/PD-L1-based therapy	2A, UCC (NCCN, 2024i)
		Cutaneous, acral	Second	20 mg qd + pembrolizumab		2A, Ⅱ (CSCO, 2023f)
		Osteosarcoma	≥Second	+ Etoposide/ifosfamide		3, Ⅱ (CSCO, 2023b)
		Renal cell carcinoma	First	20 mg qd + pembrolizumab		FDA label (EISAI, 2024)
			≥Second	18 mg qd + everolimus	Prior 1 anti-angiogenic therapy	
		Clear cell	≥Second	+ Pembrolizumab	Immuno-oncology therapy naïve	2A, ORR (NCCN, 2025b)
					Prior immuno-oncology therapy	2A, UCC (NCCN, 2025b)
		Non-clear cell	First	+ Everolimus		2A, ORR (NCCN, 2025b)
			≥Second	+ Pembrolizumab		2A, preferred (NCCN, 2025b)
		Salivary gland tumor: adenoid cystic carcinoma				2B, UCC (NCCN, 2024h)
					Symptomatic, rapidly progressing	2A, Ⅱ (CSCO, 2024c)
		Thymic carcinomas	Second			2A, preferred (NCCN, 2024t)
		Thyroid carcinoma: medullary		24 mg qd	Symptomatic or progression	2A, ORR (NCCN, 2024u); 2A, Ⅱ (*RET*- or unknown) (CSCO, 2022b)
Ramucirumab injection 100 mg; 500 mg	NMPA indication	Gastric or gastro-esophageal junction adenocarcinoma	≥Second	8 mg/kg q2w, single or + paclitaxel	Prior fluoropyrimidine- or platinum-containing chemo	NMPA label
		Hepatocellular carcinoma	≥Second	8 mg/kg q2w, single	Alpha fetoprotein ≥400 ng/mL and prior sorafenib	
	Cancer type	Colorectal cancer	≥Second	8 mg/kg q2w, + FOLFIRI	Prior bevacizumab, oxaliplatin, and fluoropyrimidine	FDA label (ELILILLY, 2024)
				+ Irinotecan	Previous therapy without irinotecan	2A, NM (NCCN, 2024e; NCCN, 2024q)
		Mesothelioma: pleural	≥Second	+ Gemcitabine		2A, ORR (NCCN, 2024L)
		Non-small-cell lung cancer	First	10 mg/kg q2w + erlotinib	*EGFR* exon 19 deletions or exon 21 (L858R) mutations	FDA label (ELILILLY, 2024)
			≥Second	10 mg/kg q3w + docetaxel	Prior platinum-based chemo or FDA-approved therapy for *EGFR* or *ALK* genomic tumor aberrations	
	Regimen	Gastric cancer [NMPA label: single or + Paclitaxel]	≥Second	+ Fluorouracil/irinotecan + Irinotecan	2A, ORR (NCCN, 2024f)	
Surufatinib capsule 50 mg; 100 mg	NMPA indication	Neuroendocrine tumor		Single	Non-functional, well-differentiated (G1, G2)	NMPA label (Hutchison, 2024)
	Cancer type	Biliary tract cancer	Second		PS ≤ 1	2B, Ⅲ (CSCO, 2023a)
		Thyroid carcinoma: medullary			Symptomatic or progression, *RET*- or unknown	2A, Ⅱ (CSCO, 2022b)

Therapy line(s)^1^: Unless otherwise mentioned, the therapy lines in this table are for palliation. NMPA, P.R.C National Medical Products Administration; FDA, U.S. Food and Drug Administration; OR, other recommended intervention; UCC, useful in certain circumstances; NM, not mentioned; TKI, tyrosine kinase inhibitor; KPS, Karnofsky Performance Status Scale; APL, acute promyelocytic leukemia; SDH, succinate dehydrogenase; ECOG, Eastern cooperative oncology group; PS, performance status; EGFR, epidermal growth factor receptor; ALK, anaplastic lymphoma kinase; HRD, homologous recombination deficiency; VEGFR, vascular endothelial growth factor receptor. As of October 2024, drugs with underlined generic names were only approved for marketing in China.

### Off-label use of vascular endothelial growth factor receptor inhibitors

The scope of vascular endothelial growth factor receptor (VEGFR) inhibitors in China includes a total of seven drugs: anlotinib, apatinib, axitinib, fruquintinib, lenvatinib, ramucirumab, and surufatinib. The predominant form of OLU among these drugs was related to indications (Table 1). OLU involving exceeding standard treatment regimens or therapy lines was relatively infrequent. Notably, there were no OLU recommendations for fruquintinib.

Among the VEGFR inhibitors, lenvatinib had the highest number of OLU recommendations for cancer types, with a total of seven uses beyond its labeled indications. This was followed by apatinib, which had six off-label recommendations for cancer types. Anlotinib, axitinib, and ramucirumab each had three off-label recommended cancer types, while surufatinib had two.

In terms of treatment regimens, anlotinib, axitinib, and ramucirumab each had one recommended regimen item that constituted OLU. Recommendations for OLU related to therapy lines were rare among VEGFR inhibitors, with axitinib being the only drug for which a recommendation transitioned subsequent lines of therapy to first-line use.

### Off-label use of multi-kinase targeted tumor anti-angiogenic drugs

The multi-kinase targeted included a total of five drugs: donafenib, pazopanib, regorafenib, sorafenib, and sunitinib.

Except for donafenib, which had no OLU recommendations, the remaining four drugs had recommendations for use beyond their approved tumor types (Table 2). Notably, pazopanib and sorafenib had off-label recommendations for seven distinct cancer types. Sunitinib followed closely, with OLU recommendations for six cancer types and nine items. Regorafenib was recommended for OLU in four cancer types, comprising eight items, and also had one OLU recommendation related to dosage.

**TABLE 2:** Off-label use of multi-kinase-targeted and other tumor anti-angiogenic inhibitors.

Drug name Dosage form Strength	Off-label type	Cancer type Subtype (molecular subtyping) [NMPA label content]	Therapy line(s)^1^	Dosage, combined agent(s)	Limiting condition	Evidence and recommendation category (reference)
Multi-kinase-targeted tumor anti-angiogenic inhibitors						
Donafenib tablet: 0.1 g	NMPA indications	Hepatocellular carcinoma	First	0.2 g bid		NMPA label (SuzhouZelgen, 2022)
		Thyroid cancer: differentiated		0.3 g bid	Iodine refractory	
Pazopanib tablet 200 mg; 400 mg	NMPA indications	Renal cell carcinoma	First			NMPA label (Novartis, 2021)
			≥Second		Prior cytokine therapy	
	Cancer type	Chondrosarcoma			Metastatic and widespread disease	2A, OR (NCCN, 2025a)
		Epithelial ovarian, fallopian tube, or primary peritoneal cancer	Recurrence			2B, OR (NCCN, 2024o)
		Gastrointestinal stromal tumor	First		*SDH*-deficient	2A, UCC (NCCN, 2024g)
			Third		Prior imatinib and sunitinib	2A, Ⅲ (CSCO, 2023d)
			≥Fifth			2A, OR (NCCN, 2024g)
		Merkel cell carcinoma			Anti-PD-(L)1-contraindicated or prior anti-PD-(L)1 monotherapy	2B, UCC (NCCN, 2024j)
		Soft tissue sarcoma	First		Ineligible for IV systemic therapy or anthracycline-based regimens	2A, UCC (NCCN, 2024s)
			≥Second		Not liposarcoma, prior chemo	FDA label (NOVARTIS, 2024)
				+ Gemcitabine		2B, UCC (NCCN, 2024s)
		Alveolar soft part sarcoma Solitary fibrous tumor				2A, preferred (NCCN, 2024s); 3, Ⅲ (CSCO, 2023b)
		Angiosarcoma				2A, OR (NCCN, 2024s)
		Chondrosarcoma			Extraskeletal myxoid	2A, preferred (NCCN, 2024s)
		Dermatofibrosarcoma protuberans			With fibrosarcomatous transformation, ineligible for IV systemic therapy or anthracycline-based regimens	2A, OR (NCCN, 2024s)
		Desmoid tumors			Aggressive fibromatosis	2A, preferred (NCCN, 2024s); 2B, Ⅱ (CSCO, 2023b)
		Epithelioid hemangioendothelioma				2A, OR (NCCN, 2024s)
		Thyroid carcinoma			Radioactive iodine unsuitable	2A, UCC (NCCN, 2024u)
		Uterine sarcoma	≥Second			2A, OR (NCCN, 2024v)
Regorafenib tablet 40 mg	NMPA indications	Colorectal cancer	Third		Prior fluoropyrimidine-, oxaliplatin- and irinotecan-based chemotherapy, an anti-VEGF therapy, and, if RAS wild-type, an anti-EGFR therapy	NMPA label (Bayer, 2021)
		Gastrointestinal stromal tumors	Third		Prior imatinib and sunitinib	
		Hepatocellular carcinoma	Second		Prior sorafenib	
	Cancer type	Biliary tract cancers	Second			2B, OR (NCCN, 2024c); 2B, Ⅱ (CSCO, 2023a)
		Bone cancer				
		Ewing sarcoma	Second			2A, OR (NCCN, 2025a)
		Pleomorphic sarcoma	Second		High-grade undifferentiated	2B, OR (NCCN, 2025a)
		Osteosarcoma	Second			1, preferred (NCCN, 2025a); 2B, Ⅱ (CSCO, 2023b)
		Glioblastoma			Adult	2A, preferred (NCCN, 2024d)
		Soft tissue sarcoma	≥Second			2A, OR (NCCN, 2024s)
			Second		Not liposarcoma	2B, Ⅲ (CSCO, 2023b)
		Angiosarcoma				2A, UCC (NCCN, 2024s)
	Dosage	Colorectal cancer [NMPA label: 160 mg PO daily on days 1–21]	Third	First cycle: 80 mg PO daily on days 1–7, followed by 120 mg PO daily on days 8–14 and 160 mg PO daily on days 15–21 Subsequent cycles: regorafenib 160 mg PO daily on days 1–21 Repeat every 28 days		2A (NCCN, 2024e; NCCN, 2024q; CSCO, 2024a)
Sorafenib tablet 200 mg	NMPA indications	Renal cell carcinoma		0.4 g bid		NMPA label (Bayer, 2018)
		Hepatocellular carcinoma				
		Thyroid cancer: differentiated				
	Cancer type	Acute myeloid leukemia (*FLT3*-ITD)	Induction	+ Azacitidine + Decitabine	Not APL, age ≥60 years, PS < 3, no major concomitant diseases, mini-mental state examination <28, short physical performance battery <9	2A, Ⅰ (CSCO, 2024d)
					Age ≥18 years; intensive induction therapy unsuitable or declines; no *IDH1* mutation	2A, UCC (NCCN, 2024a)
			Maintenance	200 mg bid d1–28 for 3 cycles, then 400 mg bid d1-28 (24 months max)	Post allogeneic hematopoietic cell transplant, in remission, history of *FLT3* mutation	2A, NM (NCCN, 2024a)
			Relapsed	+ Azacitidine + Decitabine	Not APL	2A, Ⅱ (CSCO, 2024d)
					Age ≥18 years	2A, NM (NCCN, 2024a)
		Bone cancer				
		Chordoma		Single		2A, UCC (NCCN, 2025a)
		Osteosarcoma	Second	Single		2A, preferred (NCCN, 2025a); 3, II (CSCO, 2023b)
				500 mg/d + everolimus		2B, OR (NCCN, 2025a); 3, II (CSCO, 2023b)
		High-grade undifferentiated pleomorphic sarcoma	Second	Single		2B, preferred (NCCN, 2025a)
				+ Everolimus		2B, OR (NCCN, 2025a)
		Epithelial ovarian, fallopian tube, or primary peritoneal cancer	Recurrence	+ Topotecan	Platinum-resistant	2B, Ⅲ (CSCO, 2023h)
		Gastrointestinal stromal tumors	≥Fifth	Single		2A, UCC (NCCN, 2024g) NM, NM (CSCO, 2023d)
		Head and neck cancer				
		Adenoid cystic carcinoma			Symptomatic or rapidly progressing	2A, II (CSCO, 2024c)
		Salivary gland tumors				2B, UCC (NCCN, 2024h)
		Myeloid/lymphoid neoplasms with eosinophilia	Chronic phase		*FLT3* rearrangement	2A, ORR (NCCN, 2024m)
		Soft tissue sarcoma				
		Angiosarcoma		Single		2A, UCC (NCCN, 2024s)
		Desmoid tumors		Single		1, preferred (NCCN, 2024s); 2A, Ⅰ (CSCO, 2023b)
		Solitary fibrous tumor		Single		2A, preferred (NCCN, 2024s); 3, Ⅲ (CSCO, 2023b)
Sunitinib capsule 12.5 mg; 25 mg; 37.5 mg; 50 mg	NMPA indications	Gastrointestinal stromal tumor	Second	50 mg qd q1–28 q6w	Prior or intolerance to imatinib	NMPA label (PFIZER, 2022)
		Pancreatic neuroendocrine tumors	First	37.5 mg qd	Well-differentiated, adult	
		Renal cell carcinoma	First	50 mg qd q1–28 q6w		
	Cancer type	Chordoma				2A, OR (NCCN, 2025a)
		Thymic carcinomas	Second			2A, preferred (NCCN, 2024t)
		Thyroid carcinoma			Radioactive iodine, clinical trials, or other systemic therapies unsuitable	2A, UCC (NCCN, 2024u)
		Medullary			With ectopic Cushing syndrome	2B, Ⅱ (CSCO, 2022b)
		Meningiomas				2B, OR (NCCN, 2024d)
		Myeloid/lymphoid neoplasms	Chronic phase		With eosinophilia, *FLT3* rearrangement	2A, OR (NCCN, 2024m)
		Soft tissue sarcoma				
		Alveolar soft part sarcoma Solitary fibrous tumor				2A, preferred (NCCN, 2024s); 3, Ⅲ (CSCO, 2023b)
		Angiosarcoma				2A, UCC (NCCN, 2024s)
		Chondrosarcoma			Extraskeletal myxoid	2A, OR (NCCN, 2024s)
	Therapy line	Renal cell carcinoma [NMPA label: First]	Adjuvant	50 mg qd q1–28 q6w, ≤9 cycles	High recurrent risk after nephrectomy	FDA label (CPPI, 2024)
		Gastrointestinal stromal tumor [NMPA label: Second]	Neoadjuvant or first		SDH-deficient	2A, UCC (NCCN, 2024g)
Other tumor anti-angiogenic inhibitors						
Recombinant human endostatin injection 15 mg	NMPA indications	Non-small-cell lung cancer		+ Vinorelbine/cisplatin		NMPA label (Simcere, 2020)
	Cancer type	Melanoma				
		Cutaneous or acral	1st	+ Dacarbazine ± platinum + Temozolomide ± platinum	No brain metastases	2A, Ⅰ (CSCO, 2023f)
				+ Dacarbazine ± cisplatin	Unresectable brain metastases, PS 0–2	2A, II (CSCO, 2023f)
		Mucosal		+ Dacarbazine ± platinum + Temozolomide ± platinum		2A, Ⅰ (CSCO, 2023f)
		Uveal		+ Dacarbazine ± platinum + Temozolomide ± platinum	Stage Ⅳ	2A, II (CSCO, 2023f)
		Myeloid/lymphoid neoplasms with eosinophilia	Chronic phase		*FLT3* rearrangement	2A, OR (NCCN, 2024m)
		Nasopharyngeal carcinoma	First	+ Cisplatin/gemcitabine		2B, Ⅲ (CSCO, 2023g)
		Osteosarcoma	Neoadjuvant	+ Chemo	Stage Ⅱb, limb-sparing resection unsuitable	2A, II (CSCO, 2023b)
					Stage Ⅲ	Clinician experience, II (CSCO, 2023b)
	Dosage	Melanoma [NMPA label: 7.5 mg/m^2^ d1–14 q3w]		7.5 mg/m^2^ d1–14 q4w		2A, Ⅰ (CSCO, 2023f)
		Non-small-cell lung cancer [NMPA label: 7.5 mg/m^2^ d1–14 q3w]		210 mg civ (72 h or 120 h)		NM, NM (China, 2023)

Therapy line(s)^1^: Unless otherwise mentioned, the therapy lines in this table are for palliation. NMPA, P.R.C National Medical Products Administration; FDA, U.S. Food and Drug Administration; OR, other recommended intervention; UCC, useful in certain circumstances; NM, not mentioned; TKI, tyrosine kinase inhibitor; KPS, Karnofsky Performance Status Scale; APL, acute promyelocytic leukemia; SDH, succinate dehydrogenase; ECOG, Eastern cooperative oncology group; PS, performance status; EGFR, epidermal growth factor receptor; ALK, anaplastic lymphoma kinase; VEGFR, vascular endothelial growth factor receptor. As of October 2024, drug with underlined generic name was only approved for marketing in China.

Among these five drugs, sunitinib was the only one with OLU recommendations for therapy lines, specifically for adjuvant or neoadjuvant therapies in two cancer types.

### Off-label use of other tumor anti-angiogenic inhibitors

Endostatin is a potent endogenous inhibitor of angiogenesis. It is a 20 kDa C-terminal fragment of collagen XVIII, a component of the basement membrane. Endostar, a recombinant human endostatin, was launched in China on 23 July 2006 (Han et al., 2011).

The OLU recommendations for recombinant human endostatin primarily involved indications beyond the approved cancer types and dosages. There were 4 cancer types and 11 items recommended for OLU. Additionally, there were two tumor types with OLU recommendations related to dosage.

## Discussion

AAT is an essential component of malignant tumor treatment (Liu et al., 2023). However, some AAT drugs, particularly those with limited market availability, insufficient evidence, or limited clinical experience, are frequently used off-label (Fontanals et al., 2023; Van Norman, 2023; Zarkavelis et al., 2023; Liu et al., 2024). This comprehensive narrative review shows that OLU is highly prevalent across a diverse range of tumor AAT, primarily manifesting through four distinct aspects. The use of AAT drugs beyond approved labels reflects the rapid evolution of clinical evidence and the necessity of addressing complex and diverse patient needs in oncology (Gray et al., 2009).

Based on the OLU table, the most common type of OLU is cancer types. This is also the type of OLU that clinical practice pays the most attention to. There are a total of 64 recommendations for the OLU of cancer type for 14 drugs. Although we had only included recommendations for OLU of AAT in oncology, bevacizumab has emerged as the drug most frequently recommended for OLU, with 12 cancer types and 57 documented items. Lenvatinib, pazopanib, and sorafenib also show significant OLU, mainly within expanded indications. This trend highlights the clinicians’ efforts to explore new therapeutic applications for these agents beyond their currently approved cancer types, potentially offering new hope to patients with challenging diseases (Fontanals et al., 2023; Wang et al., 2025).

In contrast, the OLUs for therapy line, regimen, and dosage were relatively sparse, with each OLU category typically involving 3–4 drugs. Furthermore, in most instances, the number of associated items was also limited. This may be attributed to our setting, wherein each OLU situation could only be assigned to one type of OLU, following a classification method that prioritizes cancer type first, then therapy line, followed by regimen, and ultimately dosage. Although the span of OLU of the therapy line is considered smaller than the cancer type, it is still important to clarify whether the earlier use of later-line treatment drugs can bring clinical benefits. In addition, there should be sufficient evidence to determine whether AAT should be continued in the subsequent line of treatment following the failure of the previous drug regimen. Modification of combination therapies involves the strategic use of AAT in conjunction with chemotherapy, immunotherapy, or other agents in treatment regimens not explicitly specified in the prescribing information. Such modifications reflect the attempts to optimize treatment efficacy by leveraging the synergistic effects of different therapeutic modalities. These combinations may provide enhanced anti-tumor activity and potentially improve patient outcomes in complex clinical scenarios. Adjustments in dosage and administration are commonly implemented based on careful assessments of treatment efficacy and patient tolerance, particularly when the drugs are used in combination with other therapies (Overbeek et al., 2023). Scholars have previously proposed a nine-category OLU classification method, which more comprehensively encompasses the content of statutory product information (Chen et al., 2024). However, since some categories have minimal relevance to OLU recommendations for AAT, we adopted a more concise four-category method in this study. Future investigations may warrant consideration of a more comprehensive OLU evaluation framework.

The lack of high-quality evidence is a significant concern (Liang and Cai, 2025) as many off-label uses are based on small studies or case reports, lacking the support of large-scale randomized controlled trials. Safety concerns arise as off-label uses may increase the risk of adverse reactions, such as hypertension, proteinuria, and bleeding. Ethical and legal issues also loom large as off-label uses can lead to medical disputes, particularly in the absence of clear informed consent (Liang and Cai, 2025). Additionally, economic burdens can be substantial as anti-angiogenic drugs are expensive, potentially increasing the financial burden on patients (Shi et al., 2024; Gordon et al., 2021). Thus, OLU should adhere to the following principles: in the absence of other effective treatment options, OLU should be applied for therapeutic purposes, prioritizing the patient’s best interests; physicians must inform patients of the specific drug, reasons, and risks associated with OLU and obtain explicit informed consent; off-label practices must be supported by safety and efficacy evidence from evidence-based medicine; medical institutions should establish management mechanisms and strictly follow off-label drug use procedures; and OLU should not be conducted for the purpose of clinical trials, scientific research, or the personal interests of medical staff (Liang and Cai, 2025).

## Limitations

Although we attempted to include the most current and up-to-date recommended evidence, readers are reminded that this is not a systematic review. The perspective on off-label recommendations presented in our review is based on authoritative guidelines and, as such, may lack the most cutting-edge research evidence. Furthermore, the tumor anti-angiogenic drugs included in this review were all products marketed in China, which may differ from those in other regions.

## Conclusion

Our work offers an updated reference for the OLU of tumor AAT and highlights the need for further exploration into specific management measures for OLU in clinical practice.
