# Supplementary material for: Off-label use catalogue of tumor anti-angiogenic drugs in China: a narrative review
Source: Front Pharmacol. 2025 Oct 14;16:1668620. doi: 10.3389/fphar.2025.1668620 (PMC12558941; doi:10.3389/fphar.2025.1668620)
Supplement: Supplementary file 1 [file Table1.docx]

**Table S1. Label version/date of tumor anti-angiogenic drugs**

|  | Drug name | Label version (year/month/date) | | | Manufacturer |
| --- | --- | --- | --- | --- | --- |
|  |  | NMPA | FDA | EMA |  |
| 1 | Anlotinib | *2024/05/08* | Not approved | Not approved | Chia Tai Tianqing |
| 2 | Apatinib | *2023/03/03* | Not approved | Not approved | Jiangsu Hengrui |
| 3 | Axitinib | *2024/08/19* | *20242/09/16* | 2021/09/28 | Pfizer |
| 4 | Bevacizumab | *2024/10/11* | *2022/09/18* | *2022/11/29* | Roche; Genetech |
| 5 | Donafenib | *2022/08/16* | Not approved | Not approved | Suzhou Zelgen |
| 6 | Fruquintinib | *2023/10/19* | 2023/08/11 | Not approved | Hutchison |
| 7 | Lenvatinib | *2022/01/29* | *2024/06/07* | 2021/09/01 | Eisai |
| 8 | Pazopanib | *2021/12/31* | 2024/01/25 | 2022/10/12 | Novartis |
| 9 | Ramucirumab | *2022/09/30* | *2022/03/22* | 2024/10/21 | Eli Lilly |
| 10 | Recombinant Human Endostatin | *2020/12/30* | Not approved | Not approved | Shandong Simcere |
| 11 | Regorafenib | *2021/07/29* | 2020/12/10 | 2021/01/01 | Bayer |
| 12 | Sorafenib | *2018/11/6* | 2023/08/28 | 2023/09/28 | Bayer |
| 13 | Sunitinib | *2022/07/29* | 2021/08/30 | 2024/04/02 | Pfizer |
| 14 | Surufatinib | *2024/01/15* | Not approved | Not approved | Hutchison |

NMPA, National Medical Products Administration of China; FDA, U. S. Food and Drug Administration; EMA, European Medicines Agency.

Label version in *italics* indicate that the product information of this version was included in the off-label use table, whereas non-italic dates indicate that the product information of this version was not included in the table after screening.
