# Supplementary material for: Off-label use catalogue of tumor anti-angiogenic drugs in China: a narrative review
Source: Front Pharmacol. 2025 Oct 14;16:1668620. doi: 10.3389/fphar.2025.1668620 (PMC12558941; doi:10.3389/fphar.2025.1668620)
Supplement: Supplementary file 2 [file Table2.docx]

**Table S2. Abstraction form**

| **Drug Name** |  | | | **Drug Dosage Form** | |  | |
| --- | --- | --- | --- | --- | --- | --- | --- |
| **Strength** |  | | | **NMPA Label Version/Date** | |  | |
| **Manufacturer** |  | | | | | | |
| **NMPA Label Indication：**  **NMPA Label Dosage and Administration：** | | | | | | | |
| **Off-label Use (OLU) Types**  1.Cancer type 2. Therapy line 3. Regimen 4. Dosage 5. On label | | | | | | | |
| **OLU Evidence/Reference Types**  1.FDA label Approved; 2. EMA label Approved; 3. NCCN guideline recommended; 4. CSCO guideline recommended;  5. NHC guideline included | | | | | | | |
| OLU types | Cancer types | Therapy lines | Regimens | Limiting conditions | Evidence and recommendation category | Reference Types | Reference |
|  |  |  |  |  |  |  |  |
|  |  |  |  |  |  |  |  |
|  |  |  |  |  |  |  |  |
|  |  |  |  |  |  |  |  |
|  |  |  |  |  |  |  |  |
|  |  |  |  |  |  |  |  |
|  |  |  |  |  |  |  |  |
|  |  |  |  |  |  |  |  |
|  |  |  |  |  |  |  |  |
|  |  |  |  |  |  |  |  |
|  |  |  |  |  |  |  |  |
|  |  |  |  |  |  |  |  |
|  |  |  |  |  |  |  |  |
|  |  |  |  |  |  |  |  |
|  |  |  |  |  |  |  |  |

**Evaluator: Evaluation Date:**
