# Supplementary material for: Off-label use catalogue of tumor anti-angiogenic drugs in China: a narrative review
Source: Front Pharmacol. 2025 Oct 14;16:1668620. doi: 10.3389/fphar.2025.1668620 (PMC12558941; doi:10.3389/fphar.2025.1668620)
Supplement: Supplementary file 4 [file Table3.docx]

**Table S3 Categories of recommendation and levels of evidence in different guidelines**

|  | **NCCN guidelines** | **CSCO Guidelines** |
| --- | --- | --- |
| **Categories of recommendation** | ​​**Preferred intervention (Preferred):** interventions with superior efficacy, safety; and, when appropriate, affordable. | **Level Ⅰ:** Class 1A evidence and some Class 2A evidence. Class 1A evidence, as well as certain Class 2A evidence with high expert consensus and good accessibility in China, as Level Ⅰ recommendations. These specifically include interventions with clear indications, strong accessibility, stable therapeutic value, and inclusion in the National Medical Insurance Drug List (basic medical insurance, work-related injury insurance, and maternity insurance). |
|  | **Other recommended intervention (OR):** interventions that may demonstrate lower efficacy, higher toxicity, rely on less mature data, or yield similar outcomes but less affordable. | **Level Ⅱ**: Class 1B evidence and some Class 2A evidence. Class 1B evidence, as well as Class 2A evidence that is less accessible in China but has strong expert consensus. Specifically, these are:  •Domestic and international randomized controlled trials (RCTs) providing high-level evidence but with poor accessibility or low cost-effectiveness;  •Measures with significant clinical benefits but higher costs may also be included as Level Ⅱ recommendations when considering potential patient benefits. |
|  | **Useful in certain circumstances (UCC)**: other interventions that may be used for selected patient subgroups (as defined by the recommendation criteria). | **Level Ⅲ**: Class 2B evidence and Class 3 evidence. Interventions that are clinically customary or have exploratory value. Although the evidence-based medical evidence is relatively limited, they are deemed acceptable by the expert panel. |
| **Categories of evidence** | **​​Category 1​​:** Based on high-level evidence, there is uniform NCCN consensus that the intervention is appropriate.​​ | **Class 1A​​:** High evidence level. Supported by rigorous meta-analyses and large RCTs. Unanimous consensus among CSCO experts (supportive votes ≥80%).  **​​Class 1B​​:** High evidence level. Supported by rigorous meta-analyses and large RCTs. Generally unanimous consensus among CSCO experts (supportive votes 60%-80%).**​​** |
|  | **Category 2A​​:** Based on lower-level evidence, there is uniform NCCN consensus that the intervention is appropriate. | **Class 2A:** Moderate evidence level. Supported by meta-analyses of moderate quality, small RCTs, large well-designed retrospective studies, and case-control studies. Unanimous consensus among CSCO experts (supportive votes ≥80%). |
|  | **Category 2B​​:** Based on low-level evidence, there is NCCN consensus is appropriate. | **Class 2B:** Moderate evidence level. Supported by meta-analyses of moderate quality, small RCTs, large well-designed retrospective studies, and case-control studies. Generally unanimous consensus among CSCO experts (supportive votes 60%-80%). |
|  | **Category 3​​:** Based on any level of evidence, the NCCN Expert Panel has significant disagreement regarding whether the intervention is appropriate. | **Class 3:** Low evidence level. Supported by uncontrolled single-arm clinical studies, case reports, and expert opinions. No consensus among CSCO experts, with significant disagreement (supportive votes <60%). |
